# Childhood Clear Cell Sarcoma of Kidney: Incidence and Survival

**DOI:** 10.3389/fped.2021.675373

**Published:** 2021-05-20

**Authors:** Hui Gao, Qi-Yuan Cheng, Qian Zhao, Long-Xiang Tao, Cheng Zhang

**Affiliations:** ^1^Department of Pediatrics, The First Affiliated Hospital of Anhui Medical University, Hefei, China; ^2^Department of Radiology, The First Affiliated Hospital of Anhui Medical University, Hefei, China; ^3^Anhui Provincial Cancer Institute, The First Affiliated Hospital of Anhui Medical University, Hefei, China

**Keywords:** clear cell sarcoma, kidney neoplasms, epidemiology, incidence, survival

## Abstract

This study is to describe current incidence of childhood clear cell sarcoma of kidney (CCSK) and to investigate the present survival of this cancer. Surveillance, Epidemiology, and End Result (SEER) data was used to identify children with CCSK and Wilms tumor (WT) aged 0–19 years in the US. Age-adjusted incidences were estimated over the decades. Age- and sex-specific epidemiology was also presented. Propensity score matching was used to balance features of CCSK and WT cases. Log rank test was used to compare survivals and Cox regression was used to evaluate independent effects of factors. The present age-adjusted incidence of childhood CCSK was 0.205 per million, which remained stable for years and ranked third in all pediatric renal tumors. The incidence rate ratios for boy and age under 4 were 3 and 21, respectively. The current 5-year overall survival (OS) rate for CCSK was 87%, which is not evidently inferior to that for WT (90%); however the outcome of CCSK was significantly poorer if both groups were well-balanced (OS rate was 86 vs. 95%). Early year of diagnosis and distant metastasis were independent survival factors. In conclusion, occurrence of CCSK remains stable over the years, with an age-adjusted incidence of 0.205 per million. Boy and age under 4 are risk factors for tumor development. CCSK currently has a favorable outcome but its nature may be more aggressive than common kidney tumor, which in turn proves efficacy of modern treatment.

## Introduction

In the US, clear cell sarcoma of kidney (CCSK) comprises ~2–4% of all primary childhood renal tumors (1–3). Clinical features and treatment protocols of CCSK have been widely studied (4–9); however the epidemiology (e.g., incidence rate) is seldom described. The database that pooled 59 population-based cancer registries in 19 European countries demonstrated an age-adjusted incidence of 0.2 per million among children aged 0–14 years during the period 1978–1997, and CCSK ranked second in all childhood renal tumors (10). During the period 1973–2005, the US national cancer registry database identified 60 CCSK cases aged 0–19 years, which ranked third following Wilms tumor (WT) and renal cell carcinoma (RCC), and the authors did not give the incidence rate (11). No evidence indicates current incidence of pediatric CCSK.

CCSK was once considered with unfavorable prognosis (1, 9); however the outcome has improved substantially in recent decades after introduction of intensive chemotherapy (6). The newest document reported a 5-year overall survival (OS) of 90% (4), and early stage patients have better survival (12). Since 5-year OS rate for WT also approximates 90% (13, 14), it seems CCSK has a similar outcome with the most common pediatric renal malignancy nowadays. This suggestion is supported by cohorts of pediatric renal tumors from the UK (15), Hong Kong (16) and Japan (17), all reporting OS rates of CCSK and WT over 90%. By contrast, several studies revealed a substantial difference between survivals of the two cancer types (3, 10, 18, 19). Of note, all these studies made imbalanced comparisons because WT cases always outnumbered CCSK and the characteristics of the two groups were apparently distinct (e.g., laterality and metastasis), which significantly affected patient survival and might distort true result.

To give the current statistics of CCSK and to make a balanced comparison of the survival between CCSK and WT among children, we query data from a large, population-based cancer registry that covers ~28% of the U.S. population (20). This study also provides information about clinical features and survival factors of CCSK in children aged 0–19 years in the US.

## Materials and Methods

### Data sources

Data for analysis was acquired from the Surveillance, Epidemiology, and End Result (SEER) Program using SEER^*^Stat version 8.3.8. (21). The SEER 9 (22), 13 (23) and 18 Registries database (20) start from 1975, 1992, and 2000 and cover about 9.4, 13.4, and 27.8% of the nationwide population, respectively. SEER 18 registries custom data also provides additional treatment information (24). In different part of the study, different database was used as follows: (1) SEER 9 (1975–2017) was used to obtain age- and sex-specific number of CCSK incident cases; (2) SEER 9 (1975–1991), SEER 13 (1992–1999) and SEER 18 (2000–2017) combined were used to describe incidence trend of CCSK; (3) SEER 18 (2000–2017) was used to describe current epidemiology of CCSK, e.g., incidence and limited duration prevalence; (4) SEER 18 custom data was used to acquire CCSK and WT patient information.

### Patient Selection

The International Classification of Diseases for Oncology (ICD-O-3) codes for CCSK and WT were C64.9 8964/3 and C64.9 8960/3, respectively. All the patients aged from birth to 19 years old. Case with unknown diagnosis confirmation was excluded. Distant metastasis, including distant lymph node or organ metastasis, was identified using documentations of the extent of disease. The detailed information was presented in Supplementary Table 1. Categories of age, tumor size, year of diagnosis were made according to usual strategy. The studies involving human participants were reviewed and approved by the Ethics Committee of the First Affiliated Hospital of Anhui Medical University. Written informed consent from the participants' legal guardian/next of kin was not required to participate in this study in accordance with the national legislation and the institutional requirements.

### Statistical Analyses

The incidence, limited duration of prevalence and mortality were calculated using SEER^*^Stat version 8.3.8 (21). Rates were per million unless otherwise indicated, and were age-adjusted to the 2000 US standard population. Categorical variables were compared using chi-square test or Fisher exact test. Weighted Log-rank test was used to compare survivals among different groups. Cox proportional hazards regression was used to incorporate multiple covariates to estimate independent influence of potential factors. *P*-values < 0.05 were considered statistical significant. Propensity score (PS) was used to match WT cases whose characteristics were similar with CCSK children. We considered sex, age at diagnosis, year of diagnosis, laterality, tumor size, radiotherapy, chemotherapy and distant metastasis as matching variables. The method was the nearest neighbor 1:1 matching with a caliper of 0.04. All the analyses were performed using R-4.0.2.

## Results

**Figure 1:**
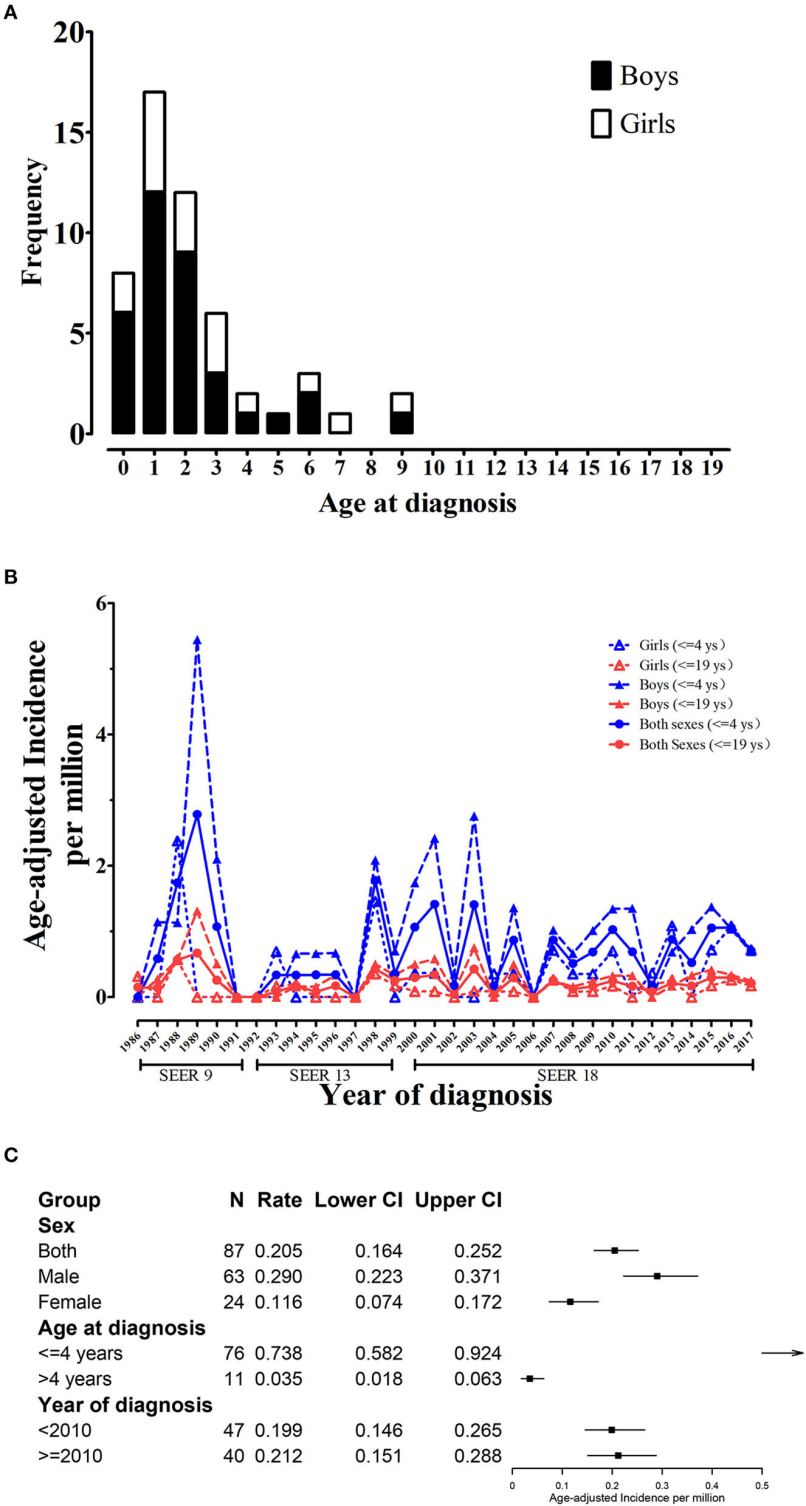
Age and sex-specific incidence trend of childhood CCSK (0–19 years). **(A)** Age and sex-specific number of childhood CCSK during 1986–2017; **(B)** Age and sex-specific trend plot of age-adjusted incidence of childhood CCSK during 1986–2017. **(C)** Group-specific age-adjusted incidence for CCSK. CCSK, clear cell sarcoma of kidney.

During 1975–2017, the SEER 9 data documented a total of 52 CCSK patients aged from 0 to 19, among whom 35 were boys and 17 were girls (M:F ratio was 2.06). Figure 1A showed CCSK cases were mostly diagnosed before 4 years, with a peak at 1 year. No case over 9 years was reported. The first CCSK child was registered in 1986 and the age-adjusted incidence fluctuated over the recent 32 years. Sex-specific trend plot showed that incidence of boys was higher than that of girls (Figure 1B). In addition, group-specific plot showed that rate of 0–4 group was higher than that of 0–19 group.

From 2000 to 2017 (SEER 18), age-adjusted incidence for CCSK aged from 0 to 19 in the US was 0.205 per million. The cumulative incidence (0–19 years) was 5.03%0. CCSK was the third common childhood renal malignancy, accounting for 3.00% of all children with kidney cancer (Supplementary Table 2). Male age-adjusted rate was 0.290, significantly higher than female (0.116, Figure 1C). The male and female cumulative incidences were 7.14 and 2.83%0, respectively. CCSK was the second and fifth common tumor among boys and girls, respectively. In addition, rate for 0–4 group (0.738) was significantly higher than that for 5–19 group (0.035, Figure 1C). CCSK ranked second among ≤ 4-year-old population (accounting for 3.95%). Among ≤ 4-year-old boys, CCSK accounted for 5.80% of all kidney cancers (Supplementary Table 2). Regarding mortality, the overall age-adjusted rate was 0.021 (95% CI was 0.010–0.041 per million), and the rates for male and female respectively were 0.037 (0.016–0.074) and 0.005 (0.001–0.027). As of January 1, 2017, 10-year limited duration age-adjusted prevalence of childhood CCSK in the US was 1.772 (per million). Likewise, 10-year prevalence for male (2.152) was higher than that for female (1.374).

**Figure 2:**
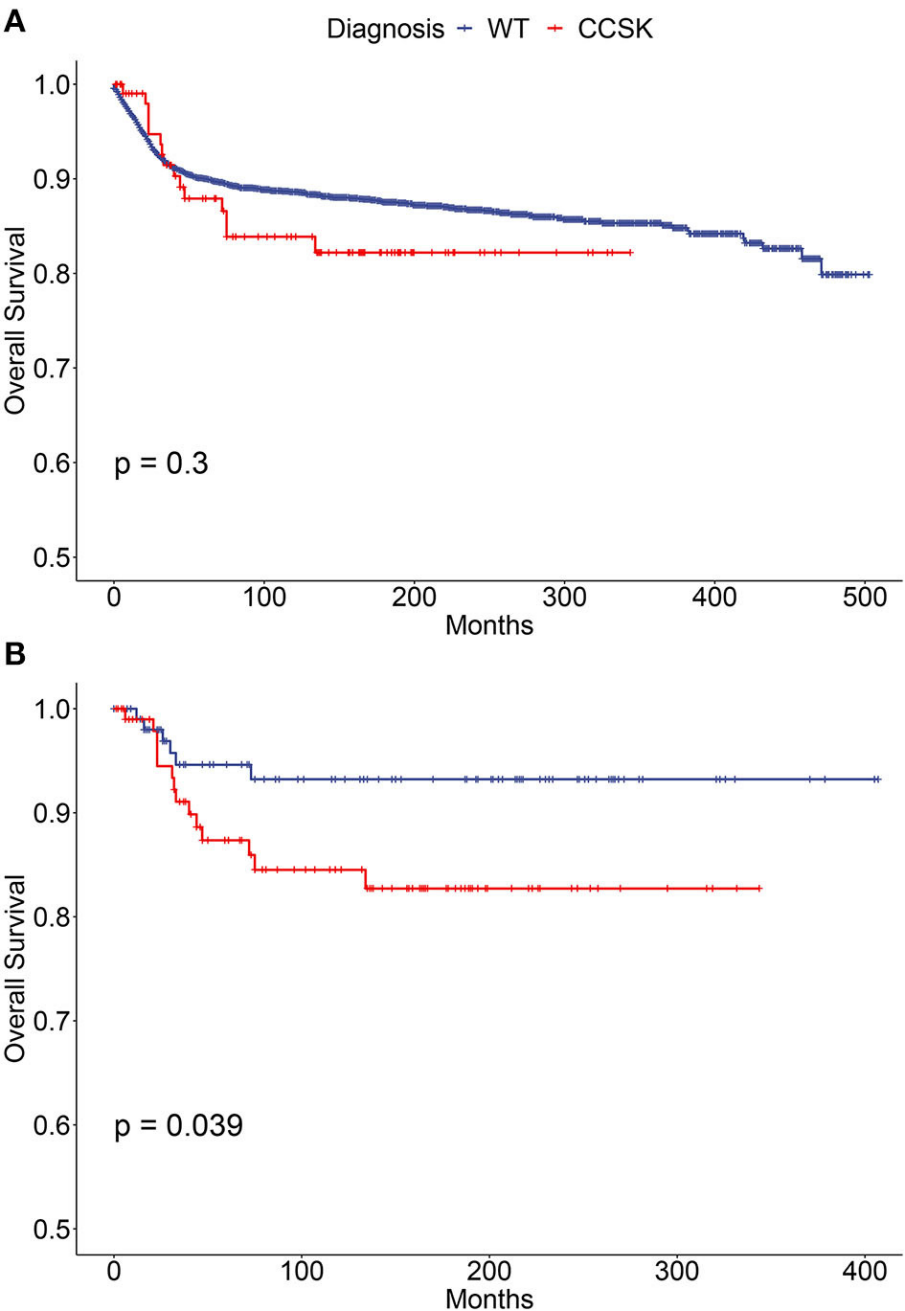
Survival curves of children (0–19 years) with WT and CCSK. **(A)** Before propensity score matching; **(B)** After propensity score matching. WT, Wilms tumor; CCSK, clear cell sarcoma of kidney.

To compare outcome of childhood CCSK with WT, CCSK (*n* = 109) and WT cases (*n* = 3 456) were indentified and features of the two groups were significantly different in many aspects (Supplementary Table 3). The survival of CCSK was reasonable (5- and 10-year rates were 86.6 and 82.6%, respectively), which were slightly worse than WT (5- and 10-year rates were 90.0 and 88.6%, respectively, Figure 2A). To improve comparability of the two cancer types, WT cases were PS matched according to characteristics of CCSK (Supplementary Table 3). In balanced comparison, the survival of CCSK was apparently worse than WT (Figure 2B). The 5- and 10-year OS rates of CCSK (85.9 and 82.7%) were substantially lower than those of WT (94.6 and 93.2%).

To study clinical characteristics of childhood CCSK, the same CCSK cohort was further analyzed. Childhood CCSK was more prone to be male predominant (M:F ratio was 2.63), occurred before 3 years old, unilateral and with large tumor size. Before 2010, 11 cases were with distant organ metastases except distant lymph nodes. After 2010, 1 case was with lung metastasis plus unknown distant lymph nodes, 1 was with bone metastasis except distant lymph nodes, and 2 were with lung and bone metastases plus unknown distant lymph nodes. No liver or brain involvement at diagnosis was recorded after 2010. Chemotherapy, radical surgery and post-operative radiotherapy were the common treatment mode. For more specific details, please see Table 1. The median follow-up time was 107 (P_25_-P_75_ was 35–189) months. Fifteen children (14%) died during the surveillance, of whom three were with distant metastases at diagnosis. The median survival time from diagnosis was 33 months. For more specific details, please see Supplementary Table 4.

**Table 1:** The clinical features and 5-year OS rates of CCSK cases aged 0 to 19 years during 1975–2016 in the US.

**Variables**	***N* (%)**	**5-year OS (95% CI)**
Total	109 (100.00)	86.6 (79.8–94.0)
**Sex**		
Female	30 (27.52)	84.1 (62.9–93.7)
Male	79 (72.48)	89.4 (79.0–94.8)
**Tumor size**		
<10 cm	24 (22.02)	94.4 (84.4–100.0)
10–15 cm	65 (59.63)	84.9 (75.8–95.2)
≥15cm	20 (18.35)	75.6 (57.2–99.9)
**Age at diagnosis**		
≤ 2 years	73 (66.97)	87.0 (79.0–95.9)
>2 years	36 (33.03)	85.8 (73.8–99.8)
**Year of diagnosis**		
<1994	14 (12.84)	71.4 (51.3–99.5)
≥1994	95 (87.16)	89.5 (82.9–96.7)
**Laterality**		
Right	48 (44.04)	93.0 (79.8–97.7)
Left	60 (55.04)	83.5 (69.6–91.4)
Bilateral	1 (0.92)	Not estimated
**Distant metastasis**		
No/Unknown	94 (86.24)	88.3 (81.4–95.8)
Yes	15 (13.76)	76.2 (55.8–100.0)
**Bone metastasis (*****n*** **=** **35)**		
No	32 (91.43)	Not estimated
Yes	3 (8.57)	Not estimated
**Lung metastasis (*****n*** **=** **35)**		
No	32 (91.43)	Not estimated
Yes	3 (8.57)	Not estimated
**Chemotherapy**		
Yes	107 (98.17)	87.6 (78.7–93.0)
No/Unknown	2 (1.83)	Not estimated
**Surgery**		
Partial nephrectomy	6 (5.51)	Not estimated
Complete nephrectomy	34 (31.19)	89.9 (79.6–100.0)
Radical nephrectomy	67 (61.47)	82.9 (73.3–93.9)
Unspecified	2 (1.83)	Not estimated
**Regional node examine (*****n*** **=** **107)**		
Yes	82 (76.64)	87.6 (79.8–96.1)
No/unknown	25 (23.36)	86.0 (72.5–100.0)
**Regional node positive (*****n*** **=** **107)**		
Yes	31 (28.97)	86.7 (73.6–100.0)
No/unknown	76 (71.03)	89.1 (81.7–97.1)
**Radiotherapy**		
After surgery	90 (82.57)	86.4 (78.9–94.6)
No/Unknown	16 (14.68)	81.2 (60.2–100.0)
Prior to surgery	3 (2.75)	66.7 (30.0–100.0)

*CCSK, clear cell sarcoma of kidney; OS, overall survival; CI, confidence interval*.

*Data source: Incidence - SEER 18 Regs Custom Data (with additional treatment fields), Nov 2018 Sub (1975–2016 varying)*.

The group-specific 5-year OS rate was also shown in Table 1. Children with tumor size no <15 cm, diagnosis before 1994, left-sided lesion and distant metastasis might have poorer outcome; however no statistical significance was detected (Supplementary Figure 1). Bilateral lesions or radiation prior to surgery might have worse outcome; however the number of sample was small (Table 1). Of note, subgroup analyses (Supplementary Figure 2) showed that children who were diagnosis before 1994 had poor outcomes if they were female, over 2 years of age, or with medium tumor size cancer. In addition, distant metastasis was a poor survival factor if children were younger than 2 years of age and with small size tumor. Multivariate analysis indicated that early year of diagnosis, large tumor size and distant metastases independently predicted worse outcome (Table 2).

**Table 2:** Multivariate analysis on potential prognostic factors for childhood CCSK.

**Variables**	**HR (95% CI)**	**P-value**
**Sex**		
Female	1 (Ref)	–
Male	0.47 (0.14–1.65)	0.240
**Tumor size**		
<10 cm	1 (Ref)	–
10–15 cm	3.35 (0.42–26.98)	0.256
≥15cm	12.33 (1.4–108.79)	0.024
**Age at diagnosis**		
≤ 2 years	1 (Ref)	–
>2 years	0.71 (0.21–2.35)	0.573
**Year of diagnosis**		
<1994	1 (Ref)	–
≥1994	0.24 (0.06–0.94)	0.041
**Distant metastases**		
No	1 (Ref)	–
Yes	5.94 (1.49–23.63)	0.012
**Radical nephrectomy**		
No	1 (Ref)	–
Yes	3.61 (0.93–14.07)	0.064

*CCSK, clear cell sarcoma of kidney; HR, hazard ratio; CI, confidence interval*.

*Data source: Incidence - SEER 18 Regs Custom Data (with additional treatment fields), Nov 2018 Sub (1975–2016 varying)*.

## Discussion

Here we demonstrate the recent age-adjusted incidence rate of pediatric CCSK is 0.205 per million children aged 0–19 in the US. So this cancer is certainly a rare cancer, that is defined as an incidence of fewer than 6 cases per 100,000 individuals per year (25). The incidence of pediatric WT is 5–6 per million (13), and in this study the number of WT children is about 30-fold more than CCSK cases, so the incidence of CCSK is also roughly estimated as 0.2 per million. The rate during 2000–2017 is similar with the European report (0.2 per million) on the period 1978–1997 (10), and the Hong Kong report (estimated as 0.24 per million) on the period 1990–2010 (16). Based on the limited information, incidence of CCSK may not be ethnically various, which is opposite to WT which incidence varies widely between ethnic groups, with Asian having the lowest rates and Black population having the highest. However, this speculation needs to be proved by international population-based studies. In addition, line plot did not show significant trend for over 30 years. Therefore, childhood CCSK occurs sporadically each year and no evidence indicates the likelihood of developing it changes in recent decades. This fact indicates genetic factors are crucial in CCSK pathogenesis.

Apart from that, male predominance and early onset of disease are well-known clinical features that also point to genetic background on CCSK oncogenesis. Here we further confirm boy and young age are two important risk factors for developing CCSK. The incidence rate ratios of boys and children ≤ 4 years are 2.5 and 21.1, respectively. The trend plot also displays the lines representing boys and children ≤ 4 are always above their counterparts. Genetic investigations on CCSK are accumulating. The National Cancer Institute has publicized the molecular characterization of CCSK in TARGET Kidney Program. It has been demonstrated that gene fusion induced by chromosome translocation and in-frame internal tandem duplication are two crucial genetic aberrations in CCSK oncology (26, 27). These molecular findings are useful for aiding histological (28, 29) and preoperative diagnosis (30), and open up for further mechanistic studies.

It is still controversial if CCSK prognosis is currently comparable with WT (3, 10, 13–19). Here the 5-year OS of WT is 3.4% higher than CCSK without statistical significance. We considered the substantial difference of clinical features, including age, metastasis, laterality, therapy mode and et al., strongly predicts survival, so we select cases with good comparability and reveal an apparently poorer outcome of CCSK. These results suggest CCSK may have similar OS with WT in real world; however the nature of this cancer may be more aggressive.

To learn more about aggressive nature of CCSK, we fully describe patient features and results are similar with literatures (7–9). However, we identify a predilection for left lesion and one case with bilateral lesions. About 4–7% CCSK cases presents with hematogenous metastases (7–9) and no true bilateral primary tumor (stage V) is reported in large collaborative studies (7, 9). Frequencies of bone and lung metastasis during 2010 to 2016 were also higher compared with other studies reporting a rate lower than 5% (4, 7, 9, 31); however this result should be treated with caution due to small sample size (*n* = 35). Feusner et al. (32) documented a striking rate of 23%, where the authors recommended both radiography and bone scanning for detection of all possible metastatic sites. Bone scan is regularly required in NWTS projects (4), but is difficult to find lesions of skull and femur, which represent over 30% of frequent sites of skeletal involvement (32). Therefore, we assume that imaging method at diagnosis could partially explain difference of metastasis rate. Brain metastasis is few at presentation (9), and our study also identifies no children with brain involvement, which is an important fact. Interestingly, brain has surpassed the bone and is now the most common site of CCSK relapse (33). This indicates that brain might be a sanctuary for cells that are protected from intensive chemotherapy; therefore brain scanning might be of value during follow-up.

Patients with large tumor size (≥15 cm), diagnosis before 1994 or distant metastasis have lower 5-year OS rates (75.6, 71.4, and 76.2%, respectively), as the overall rate is 87%. Subgroup analyses suggest some of these factors act synergistically and predict noteworthy poor outcome. Multivariate analysis further demonstrates large tumor, early diagnosis and metastasis are independent risk factors for worse OS. These results emphasize improvement of therapeutic efficacy over these decades, regardless of its malignant behavior. After 1994 in the US, NWTS-5 trial commenced and new regimen that included vincristine, doxorubicin, cyclophosphamide, and etoposide started to be used clinically (4). Better outcome appears in patients with stage I and II CCSK than NWTS-4 patients (4). Between 2006 and 2013, the AREN0321 study gave patients stage-specific chemotherapy regimen and radiotherapy, and the results will be published in the near future. Apart from the US, improved outcome over the years has been reported in several trials conducted in Europe and Japan (6). Considering CCSK has a more malignant behavior than WT and a similar outcome with WT, this study adds further support for the advancement in the treatment of pediatric CCSK. Stratification based on tumor size seems to predict survival, as shown in the survival plot; however log-rank test does not detect statistical significance. In the multivariate model, tumor size >15 cm is identified as a significant and independent factor, but the result is with wide confidence interval. Of note the NWTSG (9) and SIOP trial (7) did not identify tumor volume as a risk factor, so if large tumor indicates worse survival needs to be further investigated. Here the sample size is too small to draw a robust conclusion.

There are some limitations should be addressed. First, this study is unable to validate diagnosis from SEER database, thus selection bias may exist and accurate incidence may be influenced. Fortunately, the incidence estimated here is comparable with the aforementioned reports (10, 16). It is known that CCSK misdiagnosis happens on some occasions (7, 17), which can be reduced by pathology consultation and immunohistochemical staining. For instance, the molecular biology of CCSK involved internal tandem duplications in the BCOR gene (26), and diffuse and strong nuclear staining for BCOR was found highly specific for CCSK diagnosis (28, 29). Second, we cannot accurately stage each child due to incomplete surgical information and the extent of disease according to NWTS-5 criteria (9). Also because of incomplete data, this study fails to investigate relapse, which is not a negligible event despite current intensive chemotherapy (7, 9). Children after relapse have significantly worse survivals (33). Relapse is also an important endpoint to reflect disease outcome. It has been observed that some factors, e.g., early disease onset, influence relapse-free survival, other than OS (7, 31). So this may partly explain why younger age is not a risk factor in the present study, where OS is the only endpoint event. Besides, this study is unable to analyze the effects of chemotherapy, radiotherapy and genetic characterization, which are novel questions remain to be solved.

In conclusion, the present study demonstrates that CCSK is now the third common renal tumor among children aged 0–19 years in the US, with an age-adjusted incidence of 0.205 per million. No significant trend for incidence is observed. For boys under 4 years old, CCSK is the principle differential diagnosis for WT. CCSK currently has a favorable outcome that is similar with WT; however it has a more aggressive nature, which in turn proves the efficacy of modern therapy strategy. Prospective cooperative studies are still needed for better understanding of the risk stratification, treatment and prognosis of CCSK.

## Data Availability Statement

Publicly available datasets were analyzed in this study. This data can be found here: https://seer.cancer.gov/. Incidence - SEER Research Data, 9 Registries, Nov 2019 Sub (1975–2017) database, Incidence - SEER Research Data, 13 Registries, Nov 2019 Sub (1992–2017) database, Incidence - SEER Research Data, 18 Registries, Nov 2019 Sub (2000–2017) database, and SEER 18 Regs Custom Data (with additional treatment fields), Nov 2018 Sub (1975–2016 varying) database.

## Ethics Statement

The studies involving human participants were reviewed and approved by the Ethics Committee of the First Affiliated Hospital of Anhui Medical University. Written informed consent from the participants' legal guardian/next of kin was not required to participate in this study in accordance with the national legislation and the institutional requirements.

## Author Contributions

HG, Q-YC, QZ, and L-XT contributed to conception and design of the study. Q-YC and QZ organized the database. CZ performed the statistical analysis. HG and CZ wrote the first draft of the manuscript. L-XT wrote sections of the manuscript. All authors contributed to the article and approved the submitted version.

## Conflict of Interest

The authors declare that the research was conducted in the absence of any commercial or financial relationships that could be construed as a potential conflict of interest.
