# Supplementary material for: Childhood Clear Cell Sarcoma of Kidney: Incidence and Survival
Source: Front Pediatr. 2021 May 20;9:675373. doi: 10.3389/fped.2021.675373 (PMC8173214; doi:10.3389/fped.2021.675373)
Supplement: Supplementary file 1 [file Table_1.DOCX]

| Supplementary Table 1. Recoding rule for distant metastasis | | |
| --- | --- | --- |
| Variable name | Code | Description |
| Mets at DX-Distant LN (2016+) | NA | Yes; distant lymph node metastases |
| Mets at DX-Other (2016+) | NA | Yes; distant mets in known site(s) other than bone, brain, liver, lung, dist LN |
| SEER Combined Mets at DX-bone (2010+) | NA | Yes |
| SEER Combined Mets at DX-liver (2010+) | NA | Yes |
| SEER Combined Mets at DX-lung (2010+) | NA | Yes |
| CS Mets at DX | 10 | Distant lymph node(s) |
| (2004-2015) | 20 | Extension to:  Contralateral kidney  Contralateral ureter  Liver from left kidney  Spleen from right kidney |
|  | 40 | Noncontiguous ipsilateral adrenal (suprarenal) gland metastasis  (Contiguous involvement coded in CS Extension )  Distant metastasis except distant lymph node(s)  Carcinomatosis |
|  | 55 | Distant metastasis or extension coded in 20 plus distant lymph node(s) |
|  | 60 | Distant metastasis, NOS |
| EOD 10 – extent  (1988-2003) | 65 | Extension beyond Gerota’s fascia to:  Ureter, incl. implant(s),  ipsilateral  Tail of pancreas  Ascending colon from right  kidney  Descending colon from left  kidney  Duodenum from right kidney  Peritoneum  Diaphragm  Psoas muscle |
|  | 75 | Spleen  Liver  Stomach |
|  | 80 | FURTHER contiguous extension |
|  | 85 | Metastasis |
| EOD 10 – nodes  (1988-2003) | 7 | DISTANT Lymph Nodes  Other than above |
| EOD 4 – extent  (1983-1987) | 7 | Extension to  Adrenal gland, ipsilateral  Ureter, incl. implant(s), ipsilateral  Peritoneum  Diaphragm  Tail of pancreas  Ascending colon from right kidney  Descending colon from left kidney  Duodenum from right kidney  Ribs |
|  | 8 | FURTHER extension or metastasis  Contralateral kidney  Contralateral ureter  Contralateral adrenal gland  Stomach  Spleen  Other distant metastasis |
| EOD 4 – nodes | 5 | Contralateral or bilateral regional lymph nodes |
| (1983-1987) | 7 | Other than above (DISTANT lymph nodes) |
| NOTE: This table did not present all contents of the coding manuals. Only rules for distant metastasis in Wilms tumor and clear cell sarcoma of the kidney were included. | | |

| Supplementary Table 2. Five leading childhood kidney malignancies in the US during 2000 – 2017 | | |
| --- | --- | --- |
| **Diagnosis** | **N** | **%** |
| Both sexes | 2,900 | 100.00 |
| 8960/3: Nephroblastoma | 2,356 | 81.24 |
| 8312/3: Renal cell carcinoma | 121 | 4.17 |
| 8964/3: Clear cell sarcoma of kidney | 87 | 3.00 |
| 8963/3: Malignant rhabdoid tumor | 62 | 2.14 |
| 8310/3: Clear cell adenocarcinoma | 50 | 1.72 |
| Female | 1,524 | 100.00 |
| 8960/3: Nephroblastoma | 1,269 | 83.27 |
| 8312/3: Renal cell carcinoma | 61 | 4.00 |
| 8963/3: Malignant rhabdoid tumor | 33 | 2.17 |
| 8310/3: Clear cell adenocarcinoma | 28 | 1.84 |
| 8964/3: Clear cell sarcoma of kidney | 24 | 1.57 |
| Male | 1,376 | 100.00 |
| 8960/3: Nephroblastoma | 1,087 | 79.00 |
| 8964/3: Clear cell sarcoma of kidney | 63 | 4.58 |
| 8312/3: Renal cell carcinoma | 60 | 4.36 |
| 8963/3: Malignant rhabdoid tumor | 29 | 2.11 |
| 8310/3: Clear cell adenocarcinoma | 22 | 1.60 |
| ≤4-year-old | 1,923 | 100.00 |
| 8960/3: Nephroblastoma | 1,718 | 89.34 |
| 8964/3: Clear cell sarcoma of kidney | 76 | 3.95 |
| 8963/3: Malignant rhabdoid tumor | 61 | 3.17 |
| 9500/3: Neuroblastoma, NOS | 26 | 1.35 |
| 8312/3: Renal cell carcinoma | 6 | 0.31 |
| >4-year-old | 977 | 100.00 |
| 8960/3: Nephroblastoma | 638 | 65.30 |
| 8312/3: Renal cell carcinoma | 115 | 11.77 |
| 8310/3: Clear cell adenocarcinoma | 47 | 4.81 |
| 8260/3: Papillary adenocarcinoma | 32 | 3.28 |
| 8510/3: Medullary carcinoma | 18 | 1.84 |
| ≤4-year-old male | 948 | 100.00 |
| 8960/3: Nephroblastoma | 829 | 87.45 |
| 8964/3: Clear cell sarcoma of kidney | 55 | 5.80 |
| 8963/3: Malignant rhabdoid tumor | 28 | 2.95 |
| 9500/3: Neuroblastoma | 11 | 1.16 |
| 8312/3: Renal cell carcinoma | 4 | 0.42 |
| Data source: Incidence - SEER Research Data, 18 Registries, Nov 2019 Sub (2000-2017) | | |

| Supplementary Table 3. The characteristics of WT and CCSK before and after propensity score matching, N (%) | | | | | | | |
| --- | --- | --- | --- | --- | --- | --- | --- |
| **Group** | **Before matching** | | |  | **After matching** | | |
|  | **WT**  **(n = 3 456)** | **CCSK**  **(n = 109)** | **P-value** |  | **WT**  **(n = 105)** | **CCSK**  **(n = 105)** | **P-value** |
| Sex |  |  |  |  |  |  |  |
| Female | 1 865 (53.96) | 30 (27.52) | <0.001 |  | 29 (27.62) | 29 (27.62) | 1.000 |
| Male | 1 591 (46.04) | 79 (72.48) |  |  | 76 (72.38) | 76 (72.38) |  |
| Age at diagnosis |  |  |  |  |  |  |  |
| ≤ 2 | 1 559 (45.11) | 73 (66.97) | <0.001 |  | 69 (65.71) | 69 (65.71) | 1.000 |
| > 3 | 1 897 (54.89) | 36 (33.03) |  |  | 36 (34.29) | 36 (34.29) |  |
| Year of diagnosis |  |  |  |  |  |  |  |
| < 1994 | 824 (23.84) | 14 (12.84) | 0.008 |  | 13 (12.38) | 13 (12.38) | 1.000 |
| ≥ 1994 | 2 632 (76.16) | 95 (87.16) |  |  | 92 (87.62) | 92 (87.62) |  |
| Laterality |  |  |  |  |  |  |  |
| Unilateral | 3 212 (92.94) | 108 (99.08) | 0.013 |  | 105 (100.00) | 105 (100.00) | NA |
| Bilateral | 244 (7.06) | 1 (0.92) |  |  | 0 (0.00) | 0 (0.00) |  |
| Tumor size |  |  |  |  |  |  |  |
| < 10 cm | 1 380 (48.17) | 28 (26.17) | <0.001 |  | 28 (26.67) | 28 (26.67) | 1.000 |
| ≥ 10 cm | 1 485 (51.83) | 79 (73.83) |  |  | 77 (73.33) | 77 (73.33) |  |
| Radiotherapy |  |  |  |  |  |  |  |
| Yes | 1 579 (45.69) | 93 (85.32) | <0.001 |  | 90 (85.71) | 90 (85.71) | 1.000 |
| No/unknown | 1 877 (54.31) | 16 (14.68) |  |  | 15 (14.29) | 15 (14.29) |  |
| Chemotherapy |  |  |  |  |  |  |  |
| Yes | 3 151 (91.17) | 107 (98.17) | 0.010 |  | 103 (98.10) | 103 (98.10) | 1.000 |
| No/unknown | 305 (8.83) | 2 (1.83) |  |  | 2 (1.90) | 2 (1.90) |  |
| Distant metastasis |  |  |  |  |  |  |  |
| Yes | 755 (21.85) | 15 (13.76) | 0.057 |  | 15 (14.29) | 15 (14.29) | 1.000 |
| No/unknown | 2 701 (78.15) | 94 (86.24) |  |  | 90 (85.71) | 90 (85.71) |  |
| Abbreviation: WT, Wilms tumor; CCSK, clear cell sarcoma of kidney  Data source: Incidence - SEER 18 Regs Custom Data (with additional treatment fields), Nov 2018 Sub (1975-2016 varying) | | | | | | | |

| Supplementary Table 4. Detailed information of the 15 deceased children with CCSK. | | | | | | | |
| --- | --- | --- | --- | --- | --- | --- | --- |
| **ID** | **Survival months** | **Age at Dx** | **Race** | **Sex** | **Year of Dx** | **Tumor size (mm)** | **Distant Mets** |
| 2363037 | 33 | 6 | Black | Female | 1999 | 120 | No/Unknown |
| 14036761 | 23 | 1 | Black | Male | 2008 | 120 | Yes |
| 17992461 | 31 | 7 | White | Female | 1986 | 97 | Yes |
| 21198014 | 21 | 3 | White | Male | 1987 | 97 | No/Unknown |
| 21206309 | 72 | 1 | White | Female | 1988 | 125 | No/Unknown |
| 28955151 | 6 | 3 | White | Female | 2007 | 150 | No/Unknown |
| 30878624 | 23 | 0 | White | Male | 2001 | 125 | No/Unknown |
| 34958670 | 44 | 1 | White | Male | 1999 | 160 | No/Unknown |
| 35613296 | 32 | 1 | White | Female | 1993 | 120 | No/Unknown |
| 38816937 | 75 | 2 | White | Male | 2007 | 100 | No/Unknown |
| 38930342 | 40 | 2 | White | Male | 2009 | 125 | No/Unknown |
| 49372707 | 23 | 1 | White | Male | 2003 | 170 | No/Unknown |
| 69382942 | 134 | 2 | White | Male | 2000 | 160 | No/Unknown |
| 72682277 | 47 | 2 | White | Male | 2011 | 190 | Yes |
| 77192698 | 75 | 2 | White | Male | 2007 | Unknown | No/Unknown |
