# Supplementary figures and images for: Childhood Clear Cell Sarcoma of Kidney: Incidence and Survival
Source: Front Pediatr. 2021 May 20;9:675373. doi: 10.3389/fped.2021.675373 (PMC8173214; doi:10.3389/fped.2021.675373)

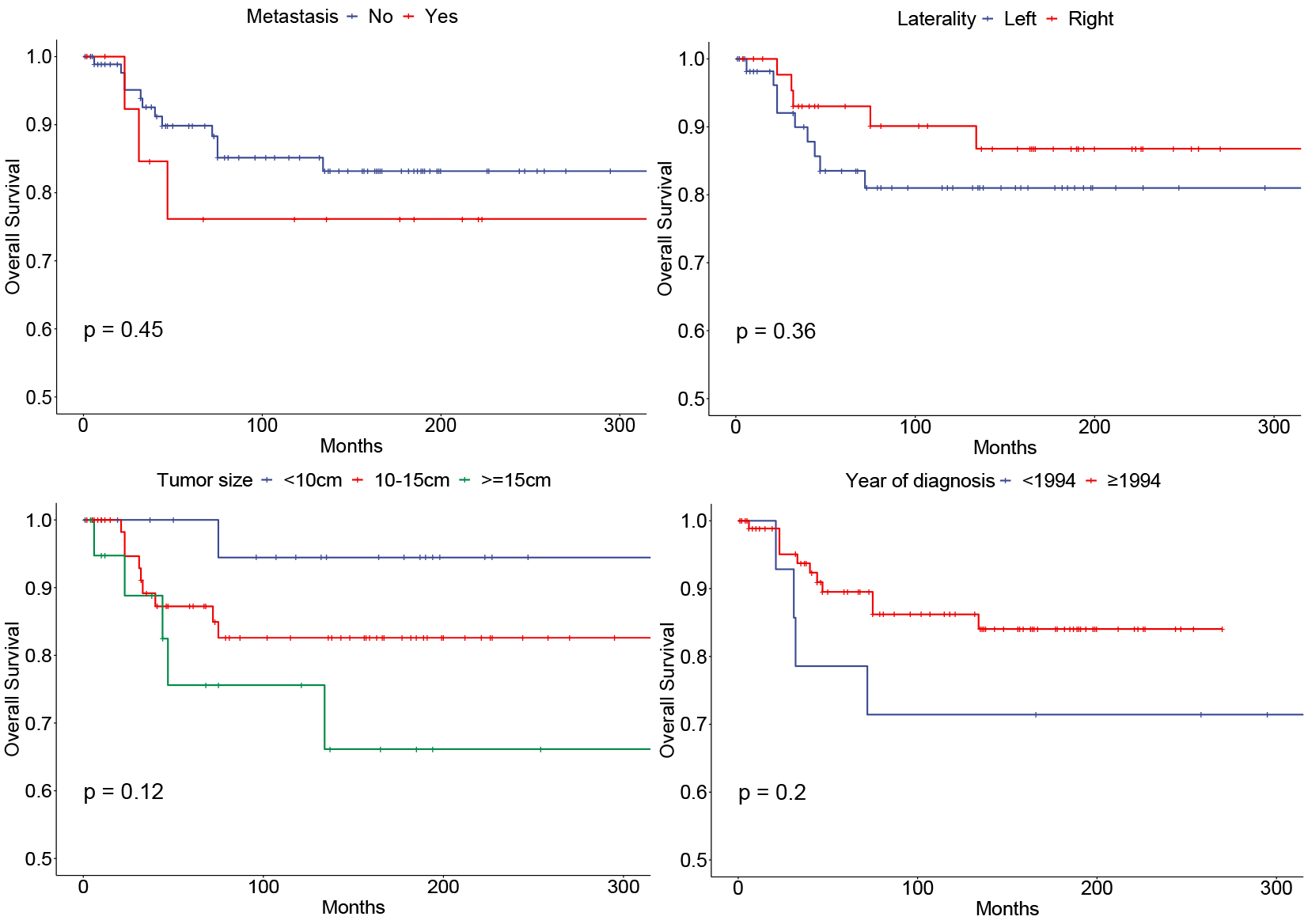

Supplement: Supplementary file 2 [file Image_1.TIF]

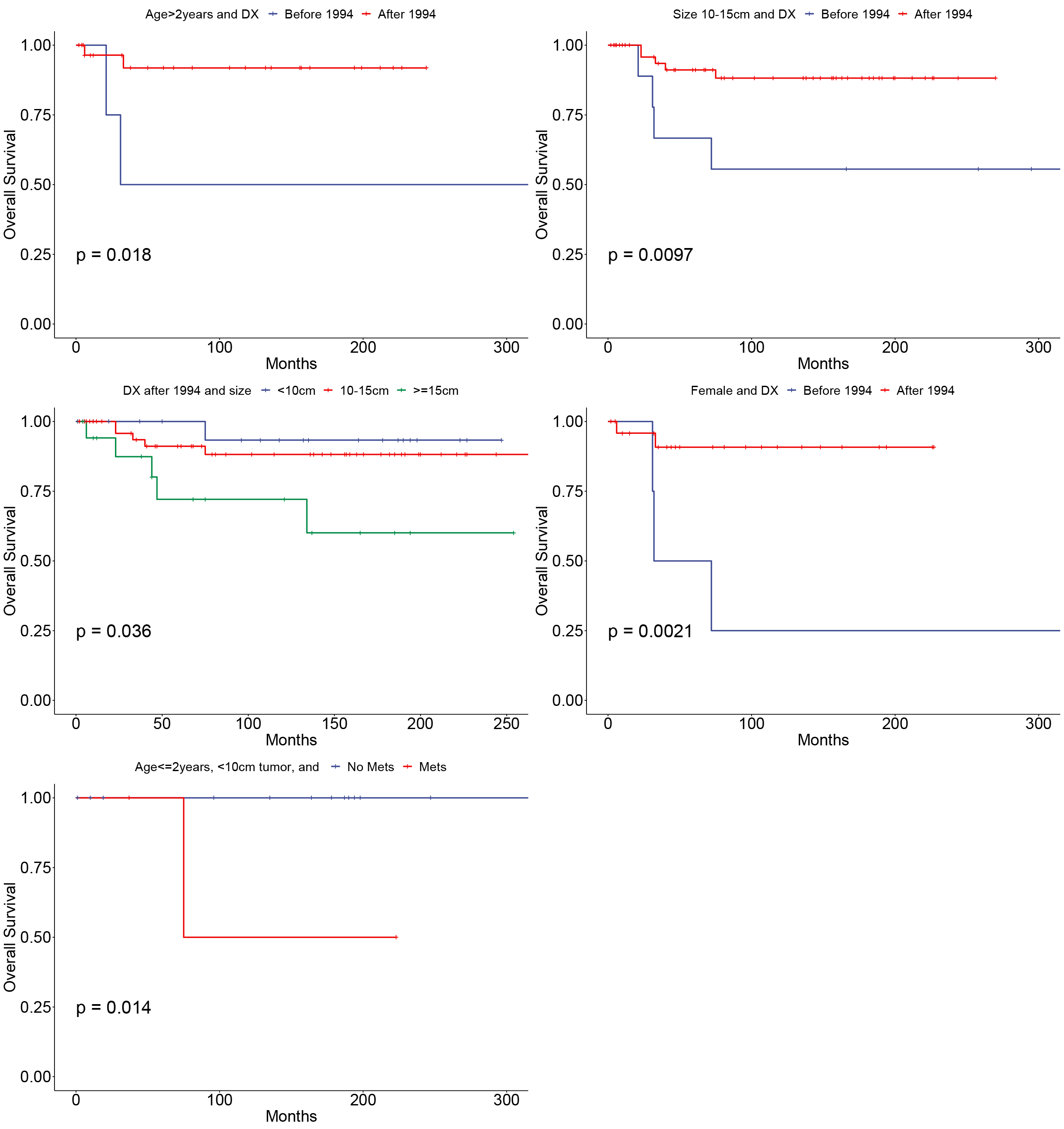

Supplement: Supplementary file 3 [file Image_2.TIF]
